# Optimization of the flow conditions in the spawning ground of the Chinese sturgeon (*Acipenser sinensis*) through Gezhouba Dam generating units

**DOI:** 10.1038/s41598-021-93903-4

**Published:** 2021-07-13

**Authors:** Anyang Huang, Jinzhong Yao, Jiazhi Zhu, Xingchen Gao, Wei Jiang

**Affiliations:** 1 Chinese Sturgeon Research Institute, China Three Gorges Corporation, JiJin Roard NO.2, Yiling District, Yichang, 443100 Hubei China; 2 Hubei Key Laboratory of Three Gorges Project for Conservation of Fishes, Yichang, China; 3 Operation and Administration Center for River Basin Hydro Complex, China Three Gorges Corporation, Yichang, China

**Keywords:** Ecology, Zoology, Ecology

## Abstract

Chinese sturgeon (*Acipenser sinensis*) is a critically endangered species, and waters downstream from Gezhouba Dam are the only known spawning ground. To optimize the velocity conditions in the spawning ground by controlling the opening mode of Gezhouba Dam generator units, a mathematical model of Chinese sturgeon spawning ground was established in FLOW-3D. The model was evaluated with velocity measurements, and the results were determined to be in good agreement. By inverting the 2016–2019 field monitoring results, the model shows that the preferred velocity range for Chinese sturgeon spawning is 0.6–1.5 m/s. Velocity fields of different opening modes of the generator units were simulated with identical discharge. The suitable-velocity area was maximal when all units of Dajiang Plant of Gezhouba Dam were open. For discharges below 12,000 m^3^/s, most of the area was suitable; for discharges above 12,000 m^3^/s, the suitable area rapidly decreased with increasing discharge. A comparison of suitable areas under high-flow showed that at discharges of 12,000–15,000 m^3^/s, opening 11–13 units on the left side was optimal. For discharges above 15,000 m^3^/s, all units should be open. We used these results to recommend a new operation scheme to support the conservation of Chinese sturgeon.

## Introduction

The Gezhouba Dam Project is the first hydropower station on the Yangtze River. The dam is intended to provide multiple benefits to society, including power generation and prevention of flooding or droughts. However, dams may also change the transport of water and sediment^1^, which affects fish habitats as in the case of the required habitats of Chinese sturgeon (*Acipenser sinensis*)^2,3^. The Chinese sturgeon is a large anadromous fish, a national first-class protection animal and a critically endangered species^4^. Before the construction of Gezhouba Dam, the spawning ground of the Chinese sturgeon were mainly in the lower reaches of the Jinsha River and upper reaches of the Yangtze River^5^. After the closure of Gezhouba Dam, the Chinese sturgeon selected a new spawning ground in the waters downstream of Gezhouba Dam, which now represents the only known Chinese sturgeon spawning ground^6^. According to the results of continuous monitoring in recent years, the reproductive geographic range and amounts of Chinese sturgeon have substantially decreased^4,7^.

Efforts to conserve the Chinese sturgeon have focused on determining suitable hydrologic or hydraulic conditions of the spawning ground. One of the hydraulic environmental variables thought to be important for reproduction is the flow velocity in the spawning habitat. A previous study has shown that the Chinese sturgeon actively selects flow velocity conditions that are beneficial to its habitat and reproduction^8^, and flow velocities exceeding the maximum tolerable velocity of the fish affect the normal habitat^9^. Research on the flow velocity of the spawning ground of the Chinese sturgeon has mainly focused on two aspects: historical data of field measurements and numerical simulation. One study based on field measurements concluded that the Chinese sturgeon chose an area with a flow velocity of 0.62–1.16 m/s when spawning^10^. The hydrological data and measured velocity of the spawning ground of the Chinese sturgeon downstream of Gezhouba Dam were analysed to calculate the velocity range of 1.0–2.0 m/s^11^. Some scholars measured the spawning days of Chinese sturgeon on site and concluded that the suitable discharge range for Chinese sturgeon was 1.07–1.65 m/s^12^. In another study, researchers measured the velocity in the spawning ground by ADCP and found that the average velocity of the spawning ground was 0.73–1.75 m/s^13^. A numerical modelling study retrieved the flow field during historical detection days and concluded that the suitable velocity range of Chinese sturgeon was 1.1–1.7 m/s^14^. In a hydrodynamic simulation of the spawning ground of Chinese sturgeon, the authors concluded that the most suitable velocity range was 0.97–1.48 m/s^15^. In another numerical modelling study, researchers simulated the water level and velocity by a two-dimensional (2D) hydraulic model and found that a velocity of 1.06–1.56 m/s was suitable for the spawning of Chinese sturgeon^16^. These results confirmed the preference of Chinese sturgeon for a range of flow velocity while spawning, but the estimated suitable ranges differ due to differences in research precision and methods. In addition, the effect of the dam operation on the characteristics of water flow in the spawning ground has been studied^17,18^, but few improvement measures and methods have been proposed. The waters downstream from Gezhouba Dam are the only known spawning ground of Chinese sturgeon, but the sensitivity of the velocity fields to the operating modes of Gezhouba Dam is unclear. The purpose of this study is to determine how to optimize releases from the dam to improve the spawning habitat.

We continuously monitored the spawning ground of Chinese sturgeon every year from 1982 during the prospective spawning period. To effectively model links from dam operations to the spawning habitat, we used a combination of numerical simulation and field monitoring. Chinese sturgeon is a type of benthic fish. After its eggs are washed away by water, they adhere to the cracks in the bottom and hatch. Therefore, the bottom hydraulic parameters are more significant than the 2D vertical average hydraulic parameters^8^. A mathematical model of the three-dimensional (3D) hydrodynamics of the spawning ground of Chinese sturgeon was established. The velocity field experienced by Chinese sturgeon from 2016 to 2019 was determined by comparing the hydroacoustic locations of fish with the model results. From this comparison, we estimated the preferred velocity range for spawning of the Chinese sturgeon. Furthermore, the optimal scheme of different generating units of Gezhouba Dam was simulated and analysed to form recommendations for alternative release procedures to support the reproduction of the sturgeon.

## Results

### Flow velocity threshold

**Figure 1:**
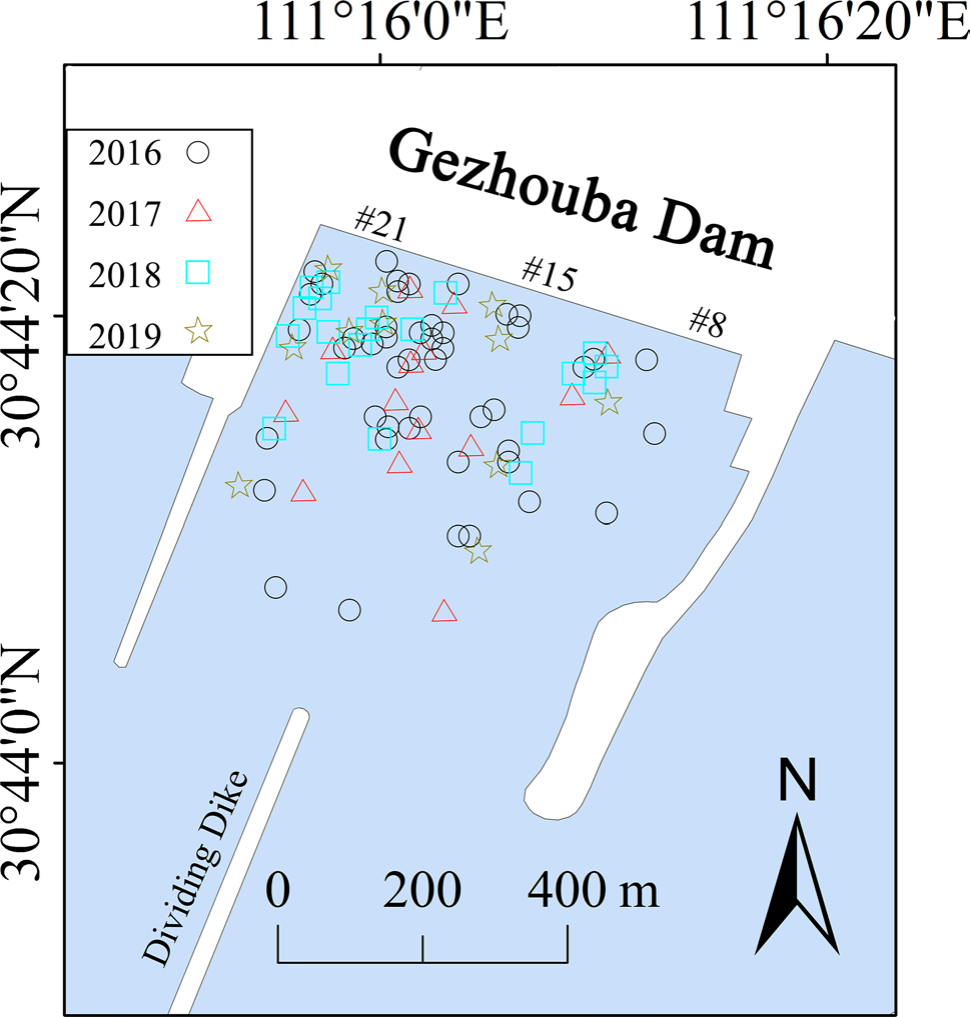
Distribution map of Chinese sturgeon signals, where ○ indicates Chinese sturgeon signals monitored in 2016, ∆ indicates those in 2017, □ indicates those in 2018, and ✩ indicates those in 2019. Map generated in ArcGIS Pro (https://www.esri.com/en-us/arcgis/products/arcgis-pro/overview).

**Figure 2:**
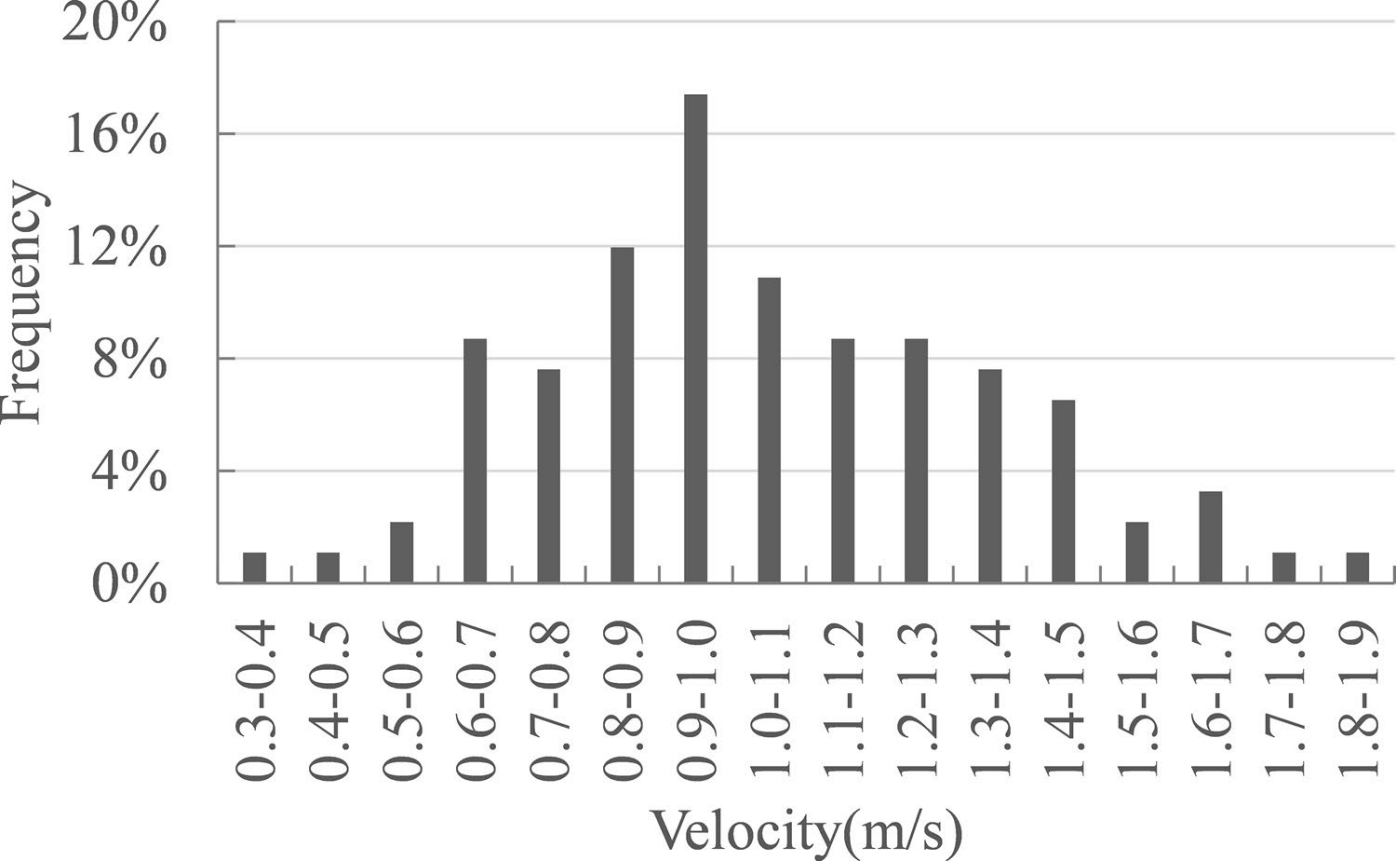
Plots of the frequency for the different flow velocity ranges of Chinese sturgeon signals.

There were 92 Chinese sturgeon signals from 2016 to 2019, which were identified with the DIDSON dual-frequency video sonar system. The distribution map of Chinese sturgeon signals was shown in Fig. 1. The number of monitored signals in 2016 was significantly higher than in 2017–2019. The latest wild reproduction of the Chinese sturgeon occurred in 2016. Overall, most Chinese sturgeon signals were distributed within 500 m downstream from Gezhouba Dam, and there were more in the right side(facing downstream) than in the left side. The flow field of each sturgeon signal was simulated by the model, and the velocity of each signal location was obtained. According to the statistical analysis of the flow velocity values, the frequency of the sturgeon signal at different flow velocity values was shown in Fig. 2. The results show that most signals were concentrated in areas with flow velocities of 0.6–1.5 m/s, which accounted for 88.1% of the signals; areas with flow velocities below 0.6 m/s accounted for 4.3% of the signals, and areas with flow velocities above 1.5 m/s accounted for 7.6%. Therefore, 0.6–1.5 m/s was selected as the preferred flow velocity range of the Chinese sturgeon for spawning activity. This result was approximately consistent with the ranges proposed by most other researchers. The low limit of the velocity range was lower than that of other researchers. There may be two reasons for this result: the first was that the bottom velocity we analysed was lower than the surface velocity and vertical average velocity under the same conditions; the second was that our research time was after 2016, and the discharge during the spawning period was relatively low, so the velocity of the Chinese sturgeon signal was also relatively low.

### Different opening modes with identical discharge

The discharge of 6150 m^3^/s on November 24, 2016, when the latest wild reproduction of Chinese sturgeon occurred, was used to study the flow velocity distribution with different opening modes. The specific opening mode cases are shown in Table 1. Case 1 was the actual situation, and the Dajiang Plant featured 7 open units: #8, #11, #13, #14, #16, #19, and #21. According to the amounts of electricity generated by Dajiang Plant and Erjiang Plant on that day, the proportion of the Dajiang River flow was 58.8%, and the average discharge of each unit was 516.6 m^3^/s. Case 2 and case 3 featured 7 open units with the same discharge, but in case 2, units #15–21 were continuously open on the right-side (facing downstream), and in case 3, units #8–14 were continuously open near the left side. Case 4 and case 5 were the most concentrated conditions with the discharge of 6150 m^3^/s because the maximum through-discharge for each unit in the Dajiang Plant is 825 m^3^/s^19^. In these cases, at least 5 units were open with an average discharge of 723 m^3^/s per unit. Case 4 involved continuously opening units #8–12 on the left side, and case 5 involved continuously opening units #17–21 on the right side. Case 6 involved simultaneously opening 14 units on Dajiang River, and the average discharge of each unit was 258.3 m^3^/s.

**Table 1:** Calculation cases with different opening modes of units under the identical discharge.

Case no	Opening mode of units	Discharge of each unit (m^3^/s)	Proportion of suitable area
1	Open 7 units according to the actual situation, #8, #11, #13, #14, #16, #19, #21	516.6	86.2%
2	Open 7 units on the left, #15–21	516.6	90.6%
3	Open 7 units on the right, #8–14	516.6	63.0%
4	Open 5 units on the right, #8–12	723	61.0%
5	Open 5 units on the left, #17–21	723	72.5%
6	Open 14 units, #8–21	258.3	95.9%

**Figure 3:**
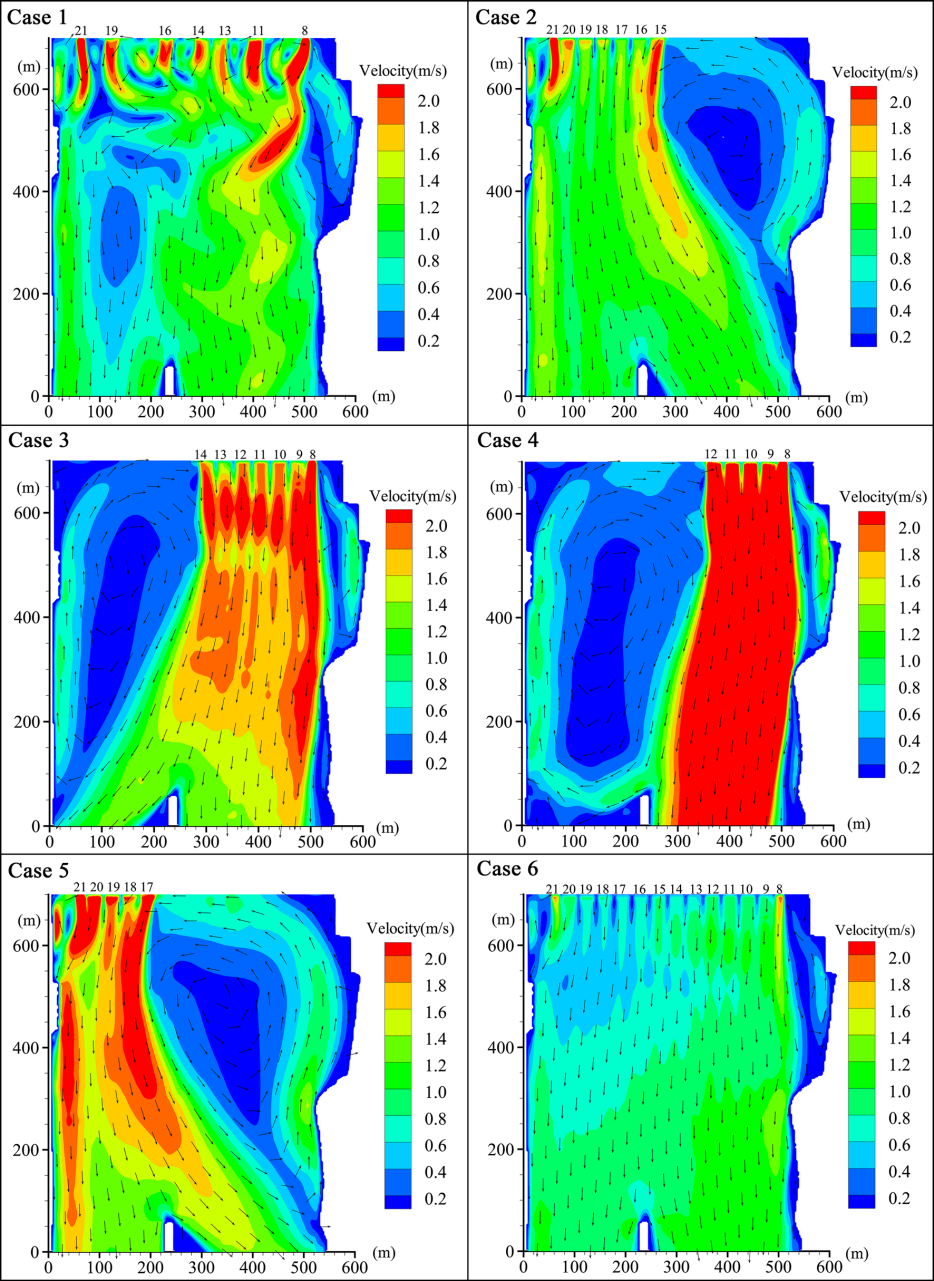
Flow field of the spawning ground in different opening modes with identical discharge, where the numbers at the top of each picture are the numbers of units to open, and the arrows indicate the direction of the water flow. Maps generated in Tecplot360 EX 2020 R1 (https://www.tecplot.com/products/tecplot-360/).

Figure 3 shows the flow fields of the spawning ground under different opening modes with identical discharge. By comparing the areas with a velocity threshold range of 0.6–1.5 m/s in different cases, the most favourable opening mode was determined. In case 1, the velocity at the outlet of the units was higher than the 1.5 m/s velocity threshold, but the discharge of each unit was only 516.6 m^3^/s, so the high-velocity range was limited, and most areas were suitable. In case 2 and case 3, there was a large difference in proportions of suitable area. Because the left side was deeper than the right side, the flow velocity on the right side was higher under the same discharge, and case 3 more easily exceeded the flow threshold, which resulted in a larger unsuitable area. Case 2 was more suitable than case 1, which also demonstrated that opening the left-side units was more favourable. In case 4 and case 5, the proportions of suitable area were small. Because the units were concentrated, the discharge of each unit was too high, and the outlet velocity was more than 2 m/s, so a large area of high velocity appeared downstream of the units with backflow under the shut-down units. The proportion of suitable area in case 5 was larger than those in case 4 and case 3, which further indicates that opening the left-side units was more favourable than opening the right-side units. Case 6 was greater than that of any other case. Because the discharge of each unit was only 258.3 m^3^/s, the velocity of the unit outlet was less than 1.5 m/s, and almost all areas were suitable except for the small areas on both sides. The suitable-velocity area was the largest when all units of the Dajiang Plant of Gezhouba Dam were open; therefore, for a given discharge, it was best to open all units.

### Different discharges under identical opening mode

The velocity distribution of the spawning field is affected by the opening mode of the units and discharge of Gezhouba Dam. To study the effect of different discharges, 14 cases were simulated, as shown in Table 2. All units of the Dajiang Plant were considered open because the proportion of suitable area was expected to be maximal under such circumstances. From 1982 to the present, the discharge during the spawning day of Chinese sturgeon under Gezhouba Dam has a wide range: the highest discharge was 27,290 m^3^/s in 1990, and the lowest discharge was 5590 m^3^/s in 2012. However, the highest design discharge of the Gezhouba Dam units is 17,930 m^3^/s^20^. Once the design discharge is exceeded, the spillway on Erjiang River discharges water, and the velocity distribution of the study area is not affected. Therefore, case 1 represents the lowest discharge of 5590 m^3^/s, and case 2 represents a discharge of 6000 m^3^/s. For each subsequent case, the discharge was increased by 1000 m^3^/s to case 13 with the highest flow of 17,930 m ^3^/s. In case 14, all units reached the design discharge, and the discharge of each unit was 825 m^3^/s^19^.

**Table 2:** Calculation cases with the same opening mode under different discharges.

Case no	Discharge (m^3^/s)	Opening mode of units	Discharge of each unit (m^3^/s)	Proportion of suitable area (%)
1	5590	Open 14 units, #8–21	243.2	95.1
2	6000	Open 14 units, #8–21	252	95.5
3	7000	Open 14 units, #8–21	294	97.1
4	8000	Open 14 units, #8–21	336	98.0
5	9000	Open 14 units, #8–21	378	97.4
6	10,000	Open 14 units, #8–21	420	94.6
7	11,000	Open 14 units, #8–21	462	96.1
8	12,000	Open 14 units, #8–21	504	70.7
9	13,000	Open 14 units, #8–21	546	66.9
10	14,000	Open 14 units, #8–21	588	65.2
11	15,000	Open 14 units, #8–21	630	54.6
11	16,000	Open 14 units, #8–21	672	40.7
12	17,000	Open 14 units, #8–21	714	26.3
13	17,930	Open 14 units, #8–21	753	20.2
14	> 17,930	Open 14 units, #8–21	825	6.0

**Figure 4:**
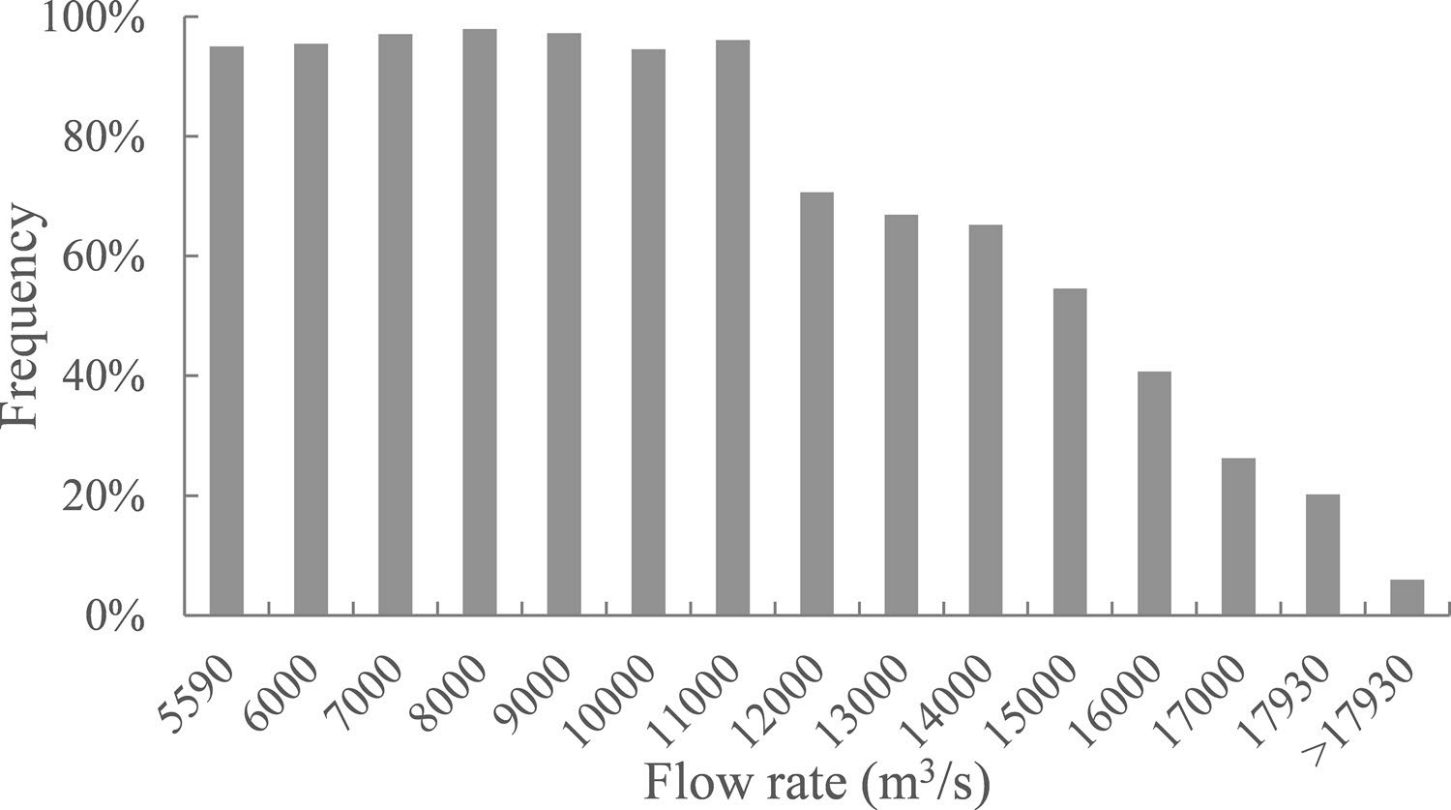
Proportions of the suitable-velocity area with all units opened under different discharges.

Figure 4 shows the proportion of suitable-velocity area with all units open under different discharges. According to the calculation results, the proportion of suitable area slightly fluctuated at approximately 96.2% for discharges of 5590–11,000 m^3^/s. Because the discharge of each unit was low, the velocity of the unit outlet was low, and most areas were within the velocity threshold. Therefore, it is advantageous to open all units when the discharge is low. After the discharge reached 12,000 m^3^/s, the proportion of suitable area rapidly decreased. Because the discharge of each unit was high, on the right side of Dajiang River, the velocity of the unit outlet exceeded the velocity threshold and increased with increases in discharge, and the range of effect gradually increased. In the last case, the proportion of suitable area was only 6% when the units reached the designed discharge of 825 m^3^/s. Because the discharge of each unit was too high, almost all areas exceeded the velocity threshold except for small areas on both sides. Therefore, at discharges below 12,000 m^3^/s, opening all units is favourable, and at discharge above 12,000 m^3^/s, a higher discharge corresponds to more unfavourable conditions.

### Optimal scheme under high-flow conditions

High-flow conditions at Gezhouba Dam are considered those that exceed 12,000 m^3^/s because of the substantive decline in suitable habitat area at higher discharges. Because opening the units on the left side of the Dajiang Plant provides a more uniform, suitable habitat, we evaluated 20 cases with a left-side opening mode under different discharge, as shown in Table 3. Because the highest discharge of each unit in the Dajiang Plant is 825 m^3^/s, at least 9 units must be open when the discharge is 12,000 m^3^/s. Case 1 was designed to open 9 units on the left, i.e., units #13–21, and the discharge of each unit was 784 m^3^/s. Cases 2–5 increased by 1 unit from left to right until 13 units were opened. For discharges of 13,000 m^3^/s, 14,000 m^3^/s, 15,000 m^3^/s, and 16,000 m^3^/s, at least 10, 10, 11, and 12 units were opened. When the discharge was 17,000 m^3^/s and 17,930 m^3^/s, at least 13 units were open.

**Table 3:** Calculation cases with different opening modes under high-flow conditions.

Case no	Discharge (m^3^/s)	Opening mode of units	Discharge of each unit (m^3^/s)	Proportion of suitable area (%)
1	12,000	Open 9 units on the left, #13–21	784.0	64.4
2	12,000	Open 10 units on the left, #12–21	705.6	75.1
3	12,000	Open 11 units on the left, #11–21	641.5	79.4
4	12,000	Open 12units on the left, #10–21	588.0	79.1
5	12,000	Open 13 units on the left, #9–21	542.8	75.5
6	13,000	Open 10 units on the left, #12–21	764.4	63.2
7	13,000	Open 11 units on the left, #11–21	694.9	72.5
8	13,000	Open 12 units on the left, #10–21	637.0	73.2
9	13,000	Open 13 units on the left, #9–21	588.0	69.9
10	14,000	Open 10 units on the left, #12–21	823.2	55.8
11	14,000	Open 11 units on the left, #11–21	748.4	64.1
12	14,000	Open 12 units on the left, #10–21	686.0	67.3
13	14,000	Open 13 units on the left, #9–21	633.2	67.3
14	15,000	Open 11 units on the left, #11–21	801.8	44.7
15	15,000	Open 12 units on the left, #10–21	735.0	46.5
16	15,000	Open 13 units on the left, #9–21	678.5	52.9
17	16,000	Open 12 units on the left, #10–21	784.0	35.2
18	16,000	Open 13 units on the left, #9–21	723.7	35.5
19	17,000	Open 13 units on the left, #9–21	768.9	23.8
20	17,930	Open 13 units on the left, #9–21	811.0	18.6

**Figure 5:**
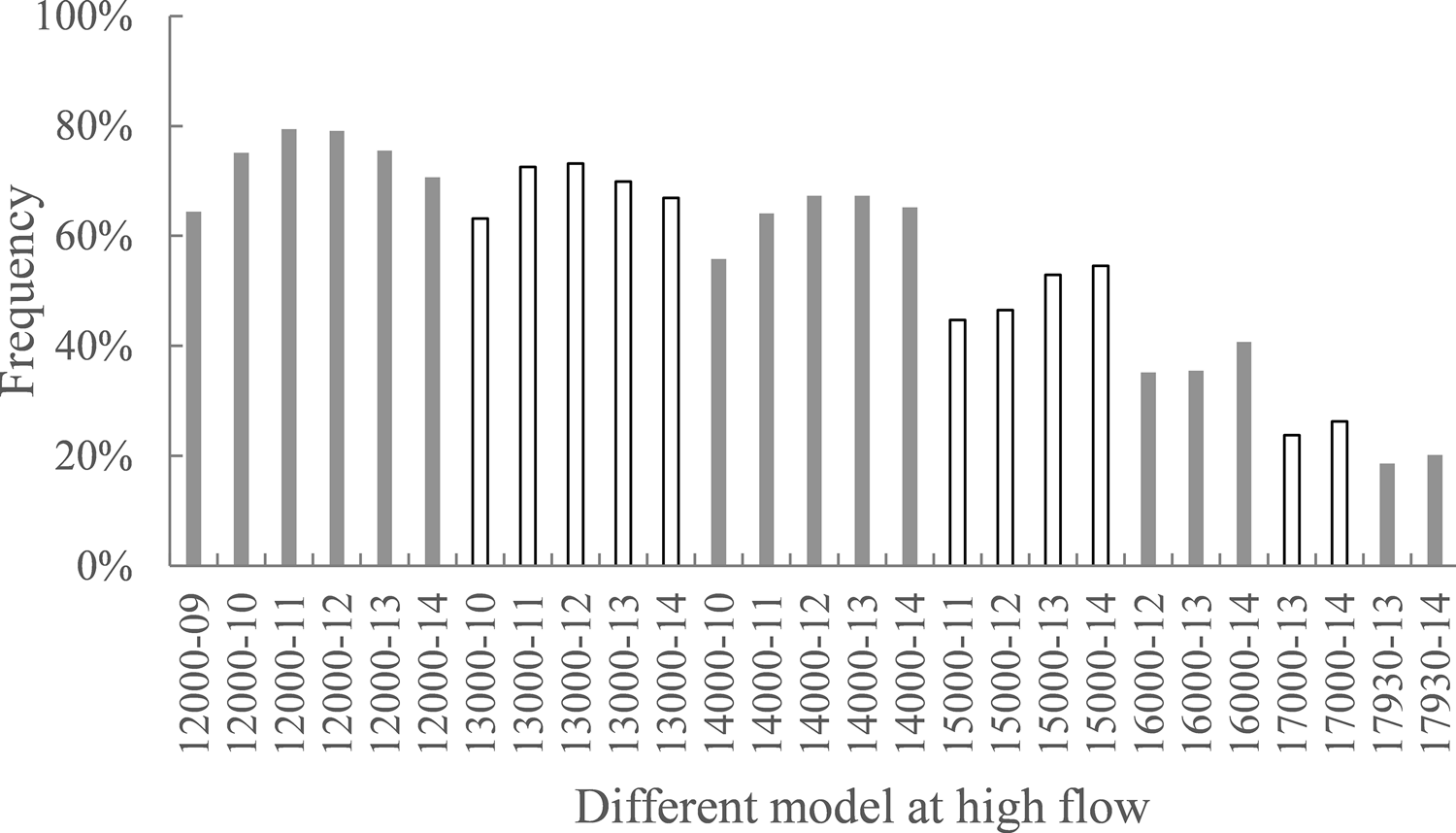
Proportions of the suitable area for different opening modes under high-flow conditions, where 12,000–09 on the x-axis indicates that the discharge is 12,000 m^3^/s, and 9 units are open on the left.

Figure 5 shows the proportions of suitable area for different opening modes under high-flow conditions. The calculation results show that when the discharge was 12,000 m^3^/s, 13,000 m^3^/s, and 14,000 m^3^/s, the proportion of suitable area showed a parabolic trend with the increase in number of units. When the discharge was 12,000 m^3^/s, the proportion of suitable area with 11 open units on the left was the largest, which was 8.7% larger than the value for all open units and 15% larger than the value for the lowest number of open units. When the discharge was 13,000 m^3^/s, 12 open units on the left had the largest proportion of suitable-flow-velocity area. When the discharge was 14,000 m^3^/s, the proportions of suitable area produced by opening 12 and 13 units on the left were the largest. The proportion of suitable area of the lowest number of open units was usually minimal because the discharge of each unit was too high, which resulted in a large area of high velocity that was not suitable for Chinese sturgeon to spawn. Because of the underwater topography, opening the left-side units was more favourable than opening the right-side units, so for all open units, the proportions of suitable area will be lower, and the number of units opened in the middle will be the most advantageous. For a discharge of 15,000 m^3^/s, with the increase in number of units, the proportion of suitable area increased, and there was no parabolic trend because the discharge of each unit exceeded 678 m^3^/s; thus, on the left side, there was a large area of high velocity, and the effect extended very far, which was not suitable for Chinese sturgeon.

## Discussion

### Spawning time and the preferred discharge of Chinese sturgeon

**Figure 6:**
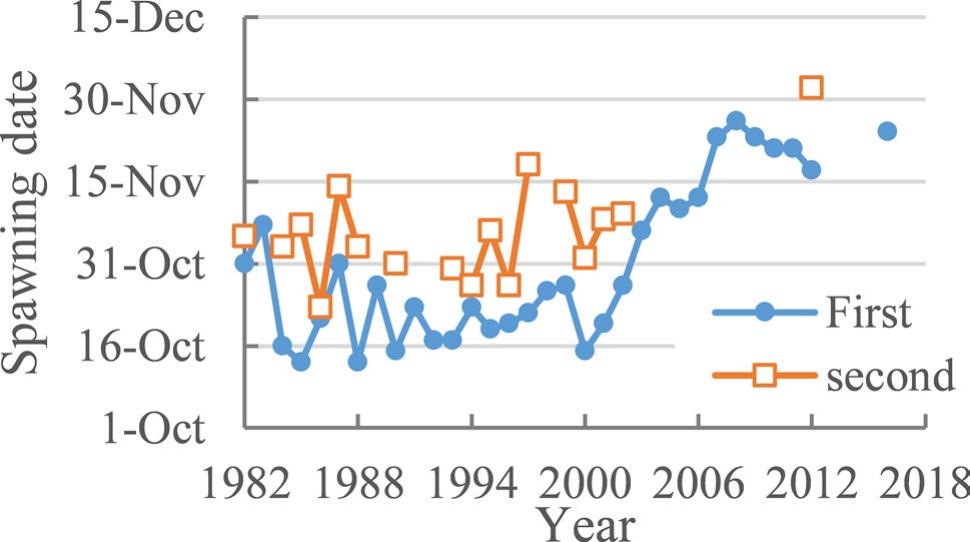
Spawning date of the Chinese Sturgeon downstream of Gezhouba Dam.

Figure 6 shows the spawning date of Chinese sturgeon downstream of Gezhouba Dam. According to the monitoring data, Chinese sturgeon spawning activity occurs 1 or 2 times every year in October–November. From 1982 to 2002, two spawning events per year were recorded, which occurred in 76.2% of the years. Since 2003, most years have featured only one spawning event, with a second spawning event occurring only once on December 2, 2012. The spawning date was mainly from mid-October to November. The first spawn was concentrated in late October before 2003, mid-November from 2003 to 2006 and late November since 2007. Therefore, the spawning date has become gradually delayed^21^. The second spawning was concentrated between late October and mid-November, generally occurring 2–27 days after the first spawning with an average of 15 days later. To date, the last spawning of Chinese sturgeon occurred on November 24, 2016, and no natural reproduction of Chinese sturgeon was observed downstream of Gezhouba Dam from 2017 to 2019.

**Figure 7:**
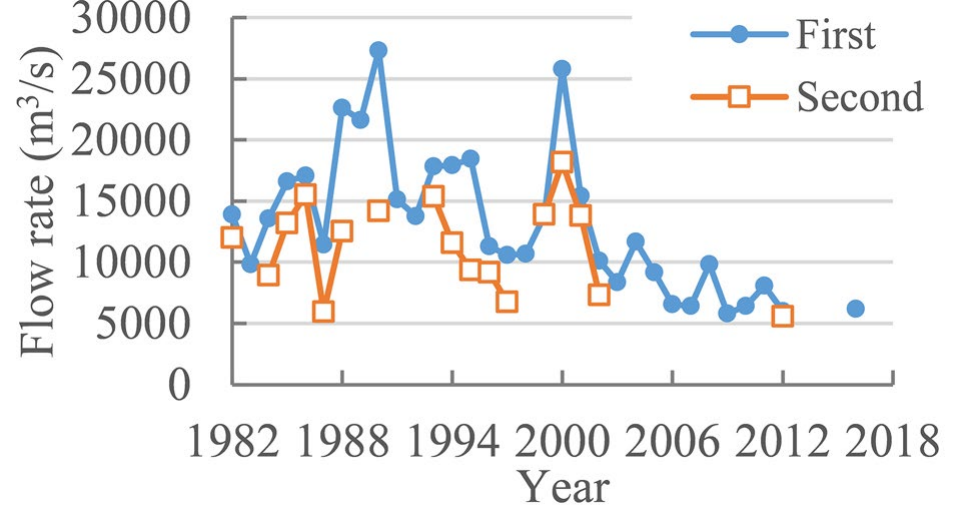
Daily discharge of the Chinese sturgeon during the spawning day downstream of Gezhouba Dam.

Figure 7 shows daily discharge of the Chinese sturgeon during the spawning day downstream of Gezhouba Dam. The highest discharge of the first spawning was 27,290 m^3^/s on October 15, 1990, and the lowest discharge was 5810 m^3^/s on November 23, 2009. The highest discharge of the second spawning was 18,170 m^3^/s on November 1, 2000, and the lowest discharge was 5590 m^3^/s on December 2, 2012. Since the spawning date gradually became delayed, the discharge of the first spawning showed a downward trend overall. The spawning discharge was less than 12,000 m^3^/s after 2002. Most spawning dates featured discharges above 12,000 m^3^/s before 2002, which accounted for 75%; discharges above 15,000 m^3^/s accounted for 55%, and discharges above the design discharge of 17,930 m^3^/s accounted for 25%. The second spawning had a lower discharge than the first spawning; discharges above 12,000 m^3^/s accounted for 52.9%, those above 15,000 m^3^/s accounted for 17.6%, and a discharge above 17,930 m^3^/s occurred only once on November 1, 2000.

### Changes in the spawning ground of Chinese sturgeon

Before the closure of Gezhouba Dam Water Conservancy in 1981, the spawning ground of the Chinese sturgeon extended from the lower reaches of Jinsha River to the upper reaches of Yangtze River, and the main spawning ground were concentrated from Pingshan to Hejiang^22^.The basic characteristics of the historical spawning ground of the Chinese sturgeon were: deep water rapids in the upstream reaches, deep backwaters in the middle reaches, and broad gravel or pebble shoals in the downstream reaches^22^.

**Figure 8:**
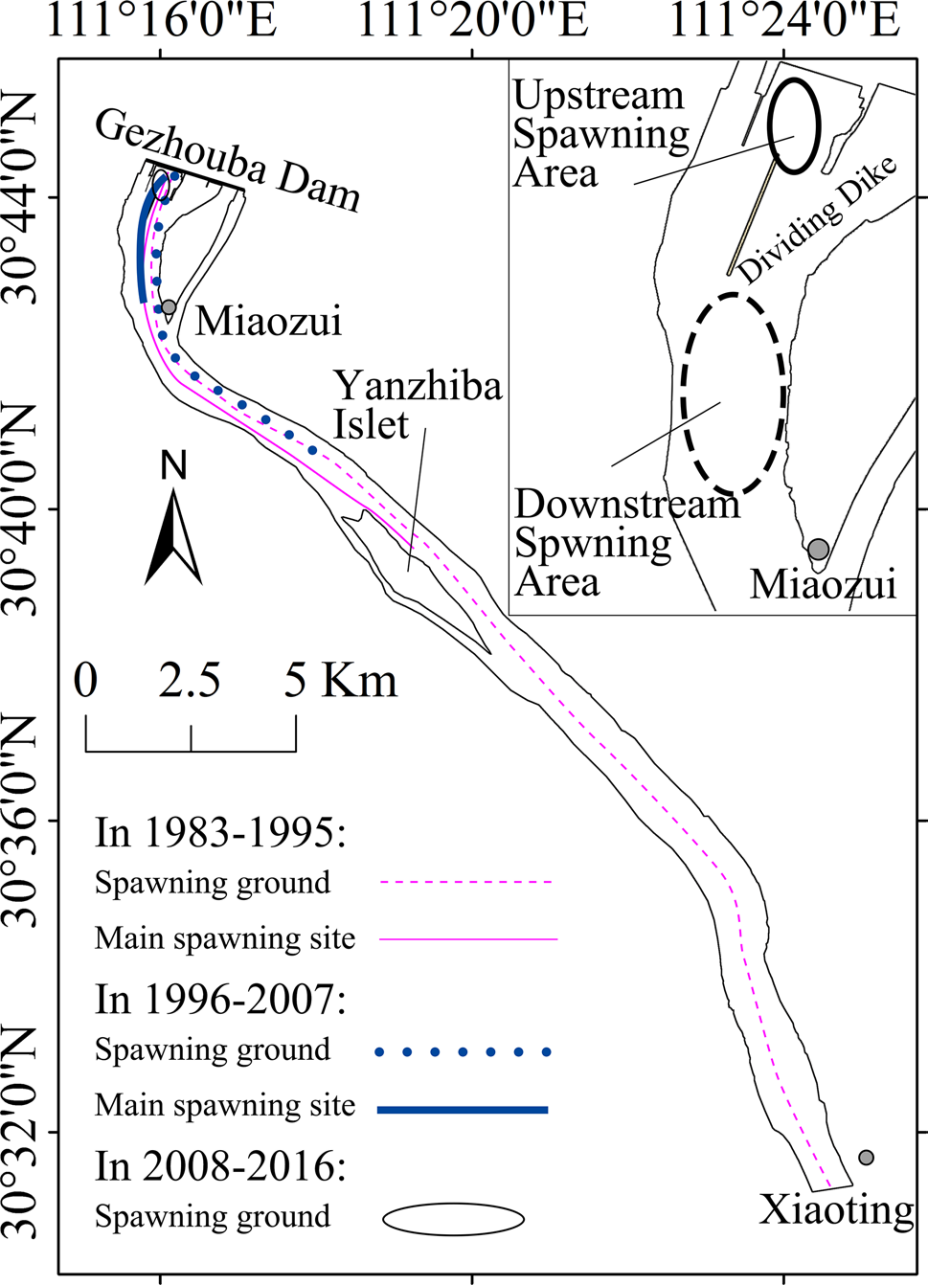
Changes in the spawning ground downstream of Gezhouba Dam of the Chinese sturgeon. Map generated in ArcGIS Pro (https://www.esri.com/en-us/arcgis/products/arcgis-pro/overview).

After the closure of Gezhouba Dam, the Chinese sturgeon selected a new spawning ground downstream of Gezhouba Dam. Many scholars studied the distribution of the new spawning ground of the Chinese sturgeon using the stomach-content analysis of egg-eating fish, ultrasonic telemetering and tracing, and egg harvesting at the bottom of the river^23–25^. The new spawning ground downstream of Gezhouba Dam shrank, as shown in Fig. 8. During 1983–1995, the range of spawning ground of the Chinese sturgeon extended approximately 30 km from Gezhouba Dam to Xiaoting, and spawning mainly concentrated in the approximately 12 km reach between Gezhouba Dam and Yanzhiba Islet^6^.

During 1996–2007, the spawning ground was the main channel of Yangtze River from Gezhouba Dam to approximately 2 km upstream of Yanzhiba Islet, and the main spawning site was within the approximately 4-km reach from Gezhouba Dam to Miaozui^25,26^. The spawning area could be divided into two parts: upstream spawning area and downstream spawning area; the spawning frequency and scale of the downstream spawning area were obviously larger than those of the upstream spawning area^12,26^. Because the spawning date was mainly concentrated in October, the spawning discharge was high, the suitable area of the upstream spawning area was small, and the upstream spawning area did not feature favourable locations for Chinese sturgeon to perch; thus, the Chinese sturgeon primarily selected to spawn in the downstream spawning area.

All natural reproduction of Chinese sturgeon has occurred in the upstream spawning area since 2008^27,28^, which was also the main research area of this paper. Since 2008, the natural reproduction date of Chinese sturgeon has been postponed to middle and late November, or even early December, when the discharges were less than 10,000 m^3^/s. The suitable area of the upstream spawning ground was large. Because Chinese sturgeon migrate as far upstream as possibly for reproduction, they selected to reproduce in the upstream spawning area. The change of spawning ground may be due to changes in discharge.

### Factors affecting spawning of the Chinese sturgeon

According to current research, the riverbed topography, bottom substrate, velocity, water temperature, water level, discharge, sediment content and other factors are thought to affect the spawning of Chinese sturgeon. Some researchers emphasized the important role of the water level^22^. Other researchers suggested that the changes in riverbed bottom substrate might have caused positional changes in the critical spawning ground of Chinese sturgeon^27,29^. Some researches indicated that the discharge and water temperatures were necessary conditions for Chinese sturgeon spawning and hatching^21^. Some researchers believed that the delay in the decrease in water temperature caused by the Three Gorges Reservoir and low numbers of reproductively mature individuals have contributed to the failure in natural breeding^30,31^.

Although many variables may contribute to the quality and quantity of the spawning habitat, we focused on the velocity as a key metric because of the direct effect of the velocity on the fish spawning habitat. In addition, the flow velocity can be managed through changes in reservoir operation and the opening mode of dam units. In contrast, the temperature, turbidity, and substrate are difficult to manipulate through management actions. The multi-variate nature of the habitat implies that velocity manipulation alone may not be sufficient, and the operation would not be effective unless combined with water temperature and sediment factors, so other factors should also be evaluated in the future because they may work together.

## Conclusions

Based on the field monitoring results of 2016–2019, the FLOW-3D model was used to simulate the flow field of monitored sturgeon signals, and it was concluded that the preferred velocity range for Chinese sturgeon was 0.6–1.5 m/s. Under a given discharge, the suitable-velocity area was maximal when all units of the Dajiang Plant of Gezhouba Dam were open, and the conditions were more favourable when the units on the left side were open. When the discharge was less than 12,000 m^3^/s, the proportion of suitable area slightly fluctuated at approximately 96.2%; when the discharge was 12,000 m^3^/s, the suitable area rapidly decreased with increasing discharge. Moreover, for different opening modes at high flows, at discharges of 12,000–13,000 m^3^/s, opening 11–12 units on the left side was the best; at a discharge of 14,000 m^3^/s, opening 12–13 units on the left side was the best; when the discharge reached 15,000 m^3^/s, opening 14 units was the best. The optimal scheme for the opening mode of the units at different discharges was analysed, and the results provide new ideas for the protection and ecology conservation of Chinese sturgeon.

## Methods

### Study area

**Figure 9:**
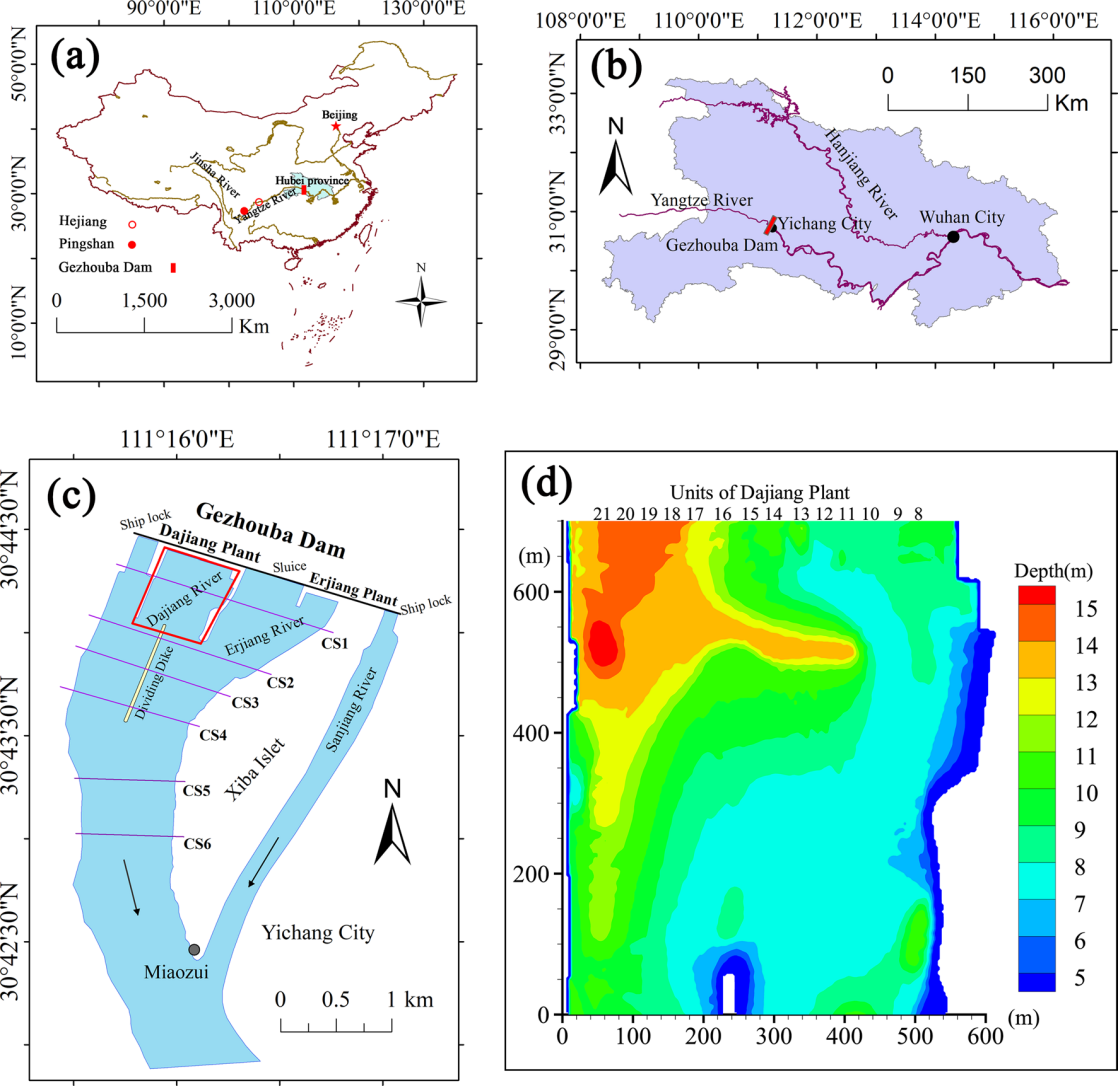
Location of the study area. (**a**) Location of Yangtze River and Hubei Province in China; (**b**) Location of Gezhouba Dam in Hubei Province, which is shown in red; (**c**) General condition of the field survey and location of the cross-sections (CS1–CS6); (**d**) Location of the units of Dajiang Plant and bathymetric map of the study area. Maps in figure (**a**), (**b**) and (**c**) generated in ArcGIS Pro (https://www.esri.com/en-us/arcgis/products/arcgis-pro/overview). Map in figure (**d**) generated in Tecplot360 EX 2020 R1 (https://www.tecplot.com/products/tecplot-360/).

Field surveys have shown that the only known spawning ground is located in the section between Gezhouba Dam and Miaozui, which is approximately 4 km downstream of Gezhouba Dam^12^. Therefore, the area between Gezhouba Dam and Miaozui was selected for investigation in this study, as shown in Fig. 9. Figure 9a shows the location of Yangtze River and Hubei Province in China; Fig. 9b shows the location of Gezhouba Dam in Hubei Province; Fig. 9c shows general condition of spawning ground downstream Gezhouba Dam. The Gezhouba Dam Project have 21 generator units; 14 units with a capacity of 125,000 kilowatts are installed in the Dajiang Plant; 2 units with a capacity of 170,000 kilowatts and 5 units with a capacity of 125,000 kilowatts are installed in the Erjiang Plant^20^. The units are sequentially numbered from the left bank to the right bank (facing downstream). The numbers of Erjiang Plant units are #1–7, and the numbers of Dajiang Plant units are #8–21.

This area was divided into several cross-sections (Fig. 9c), and the velocity of the cross-section was measured with a 300-kHz acoustic Doppler velocity profiler (ADCP). In addition, acoustic imaging sonar monitoring was performed in this area. According to the monitoring results from 2016 to 2019, most of the Chinese sturgeon signals appeared within 700 m below the Dajiang Plant units of Gezhouba Dam, as shown in the red box in Fig. 1c. Moreover, according to the field investigation, the area of the spawning ground further decreased in recent years because most of the spawning behaviour of Chinese sturgeon has occurred in the red box in Fig. 9c since 2008^27,28^. Hence, the range of 700 m downstream of the Dajiang Plant units was the key area in this study. This area is shown in Fig. 9d, which shows a bathymetric map of this area, and the colour shading and contours represent the water depth when the water level downstream from the dam was 41.2 m. The units on the right side are #8–15, and the corresponding water area under the units was shallow, mostly 7–10 m. There is a deep pit 200 m from Gezhouba Dam with a water depth of approximately 13 m. Units #16–21 are on the left side, and the corresponding water area is deeper, i.e., 12–15 m within 300 m of the dam; then, the water depth becomes shallower to approximately 10 m.

### Numerical model

In this study, we used numerical model FLOW-3D, which is a commercial CFD package based on the finite volume method (FVM) that solves the Reynolds-averaged Navier–Stokes equations^32^. It can effectively estimate the flow structure and velocity distribution in different water layers^33^.

#### Boundary and initial conditions

The upstream boundary condition used the known discharge based on releases from Gezhouba Dam. The pressure boundary was used for the dowstream boundary and set to the water level. The water surface was a free surface, using a pressure boundary, given standard atmospheric pressure. The wall boundary was used for the solid boundary of the bottom and both sides. The initial condition was the water level, and the initial velocity was 0.

#### Mesh construction

A hexahedral orthogonal grid was used to mesh the model, which can iteratively define a base mesh to fit the surface geometries. The finite volume method was used to discretize the governing equation, and the GMRES algorithm was used to solve the equations^34^. The mesh sizes were selected to respect the requirements of the grid convergence index (GCI) method to test the spatial convergence^35^. The X-axis direction and Y-axis direction mesh sizes were 3–8 m, and the Z-axis direction mesh sizes were 1–2 m.

#### Model evaluation

**Figure 10:**
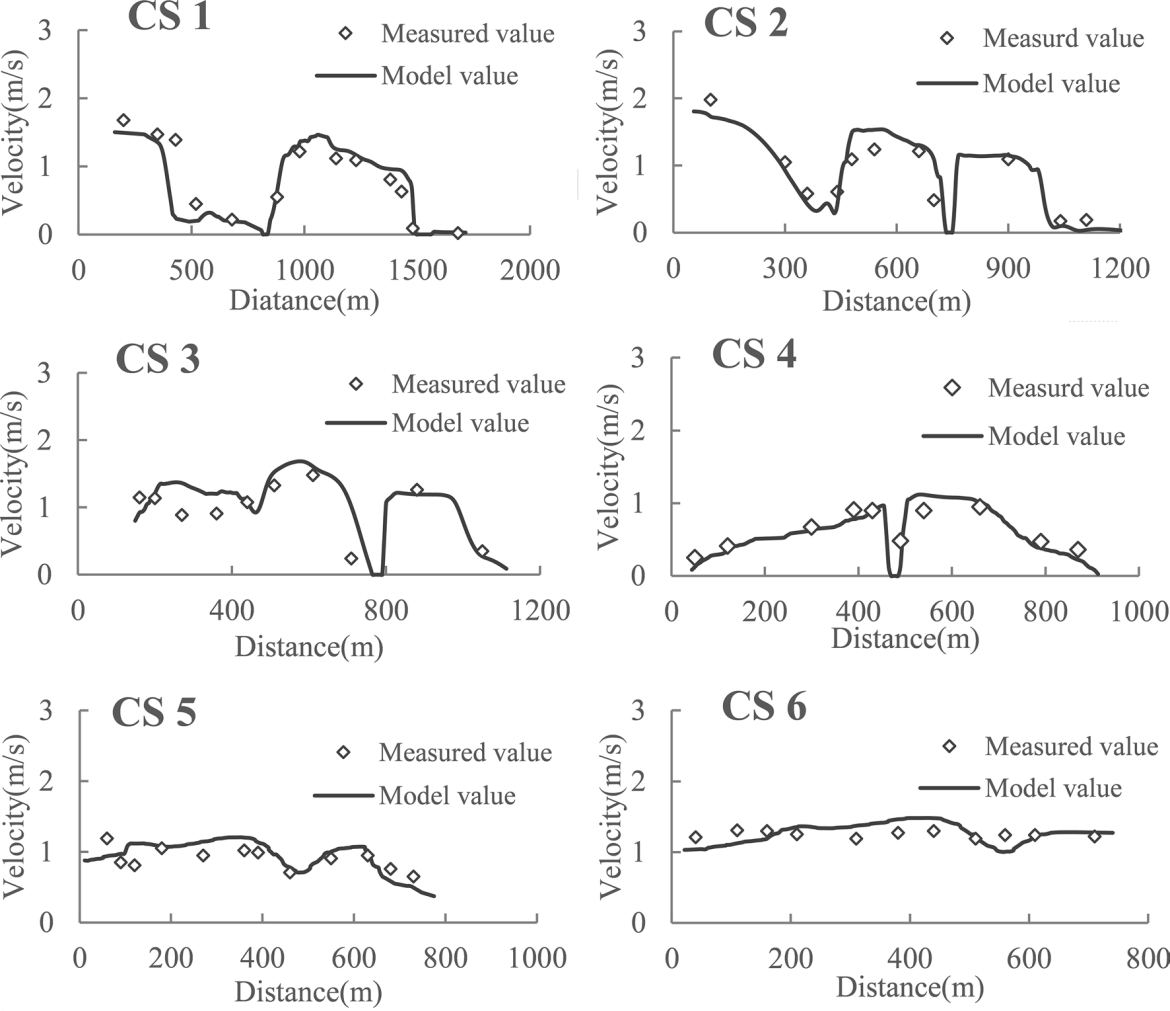
Plots of the measured and model values for cross-sections 1–6 (Fig. 9c).

The measured velocity data from downstream of Gezhouba Dam were used to evaluate the model. The discharge of Gezhouba Dam was 12,000 m^3^/s, and the water level downstream from the dam was 41.2 m. The comparisons of the bottom velocity of the measured and model values for cross-sections 1–6 are presented in Fig. 10. From Fig. 10, the distribution of flow velocity of each cross-section was in good agreement, especially in the Dajiang River area, where the latest spawning ground of the Chinese sturgeon was located. The errors of the model and measured values were generally less than 0.2 m/s, and the maximum error was 0.43 m/s, which appeared next to the dividing dike in cross-section 3. The two-tailed t-test permutation of the model and measured values showed no significant difference: P = 0.45 > 0.05. Therefore, the model simulation was reasonable and acceptably simulated the water flow characteristics of the spawning ground of Chinese sturgeon.

### Acoustic monitoring

Acoustic imaging sonar is a fast and effective method to study Chinese sturgeon because it can monitor the number and distribution of fish without approaching and harming the fish^36^. For acoustic monitoring, this paper used a Dual-Frequency Identification Sonar (DIDSON) system, which is widely used in fishery management, structural detection, pipeline leakage identification, underwater monitoring, underwater searching, underwater security inspection, etc.^37^.

The main monitoring area was approximately 4 km long between Gezhouba Dam and Miaozui. During the investigation, the sonar transmitter was fixed to the side of the survey vessel and located 0.3 m below the water surface. A GPS device produced by the Garmin company was used for navigation and positioning. We continuously performed monitoring every day from October to January of the following year for 3–4 h with a zigzag survey pattern to ensure full coverage of the spawning ground. The monitoring results were saved in the form of video images by acquisition software (DIDSON V5.25), and Chinese sturgeon signals were confirmed by measuring the full length, swimming behaviour, and body shape. To reduce the error of judgement and obtain high-accuracy Chinese sturgeon signals, each monitoring signal was confirmed by at least two different researchers.

## Data Availability

Data are available from the corresponding author upon request.
